# Differential Regulation of *Zfp30* Expression in Murine Airway Epithelia Through Altered Binding of ZFP148 to rs51434084

**DOI:** 10.1534/g3.117.300507

**Published:** 2017-12-13

**Authors:** Lucas T. Laudermilk, Joseph M. Thomas, Samir N. Kelada

**Affiliations:** * Department of Genetics, University of North Carolina at Chapel Hill, North Carolina 27599; † Curriculum in Genetics and Molecular Biology, University of North Carolina at Chapel Hill, North Carolina 27599; ‡ Marsico Lung Institute, University of North Carolina at Chapel Hill, North Carolina 27599

**Keywords:** *Zfp30*, causal variant, CXCL1, eQTL, neutrophil, ZFP148

## Abstract

Neutrophil chemotaxis to the airways is a key aspect of host response to microbes and a feature of multiple pulmonary diseases including asthma. Tight regulation of this recruitment is critical to prevent unwanted host tissue damage and inflammation. Using a mouse (*Mus musculus*) model of asthma applied to the Collaborative Cross population, we previously identified a lung gene expression quantitative trait locus (eQTL) for Zinc finger protein 30 (*Zfp30*) that was also a QTL for neutrophil recruitment and the hallmark neutrophil chemokine CXCL1. The *Zfp30* eQTL is defined by three functionally distinct haplotypes. In this study, we searched for causal genetic variants that underlie the *Zfp30* eQTL to gain a better understanding of this candidate repressor’s regulation. First, we identified a putative regulatory region spanning 500 bp upstream of *Zfp30*, which contains 10 SNPs that form five haplotypes. In reporter gene assays *in vitro*, these haplotypes recapitulated the three previously identified *in vivo* expression patterns. Second, using site-directed mutagenesis followed by reporter gene assays, we identified a single variant, rs51434084, which explained the majority of variation in expression between two out of three haplotype groups. Finally, using a combination of *in silico* predictions and electrophoretic mobility shift assays, we identified ZFP148 as a transcription factor that differentially binds to the *Zfp30* promoter region harboring rs51434084. In conclusion, we provide evidence in support of rs51434084 being a causal variant for the *Zfp30* eQTL, and have identified a mechanism by which this variant alters *Zfp30* expression, namely differential binding of ZFP148.

Recruitment of neutrophils to the lung is a hallmark of innate immune responses to inhaled pathogens and air pollutants, and is associated with decreased lung function and disease susceptibility (Nathan 2006; Borregaard 2010; Mantovani *et al.* 2011). Neutrophils play an integral role in immune response to external stimuli by migrating toward resulting chemotactic signals and responding to pathogens through phagocytosis and secretion of reactive oxygen species, proteases, and cytokines. These reactive oxygen species and proteases, however, do not distinguish between host and pathogen, therefore a robust recruitment of neutrophils can adversely impact host tissue. This makes tight regulation of neutrophil recruitment and their activity crucial. A neutrophilic infiltrate in the airways is seen in a range of pulmonary diseases, including asthma (Jatakanon *et al.* 1999; Wenzel *et al.* 1999), acute respiratory distress syndrome (ARDS) (Chollet-Martin *et al.* 1992; Grommes and Soehnlein 2011; Williams and Chambers 2014; Kangelaris *et al.* 2015), chronic obstructive pulmonary disease (COPD) (Qiu *et al.* 2003; Quint and Wedzicha 2007; Hoenderdos and Condliffe 2013; Günay *et al.* 2014; Duman *et al.* 2015; Grabcanovic-Musija *et al.* 2015), and cystic fibrosis (Mackerness *et al.* 2008; Gifford and Chalmers 2014; Laval *et al.* 2016). In asthma, neutrophil levels in sputum have been shown to correlate with disease severity and lung function, as well as a lack of responsiveness to glucocorticoids (Stănescu *et al.* 1996; Green *et al.* 2002; Uddin *et al.* 2010).

Previously, we applied a house dust mite model of asthma to incipient lines of the Collaborative Cross (CC) population to identify novel genes and pathways associated with neutrophil recruitment responses in the context of allergic inflammation. The CC is a new mouse genetics reference population composed of recombinant inbred lines that are derived from eight founder strains (Collaborative Cross Consortium 2012). We measured neutrophil recruitment responses and levels of the hallmark neutrophil chemokine CXCL1 (aka KC) in bronchoalveolar lavage fluid and found that both were regulated by a locus on chromosome 7 (25.6–29.7 Mb) that we called *Dpc1* (Rutledge *et al.* 2014). Likewise, the expression of the gene Zinc finger protein 30 (*Zfp30*) was also regulated by this locus [*i.e.*, is an expression quantitative trait locus (eQTL)], and *Zfp30* expression was strongly correlated with CXCL1 and neutrophil counts (Supplemental Material, Figure S1). We also found that *Zfp30* is expressed in airway epithelia and that knockdown of *Zfp30* in an epithelial cell line resulted in increased CXCL1 production in response to endotoxin. These results led to development of a model in which ZFP30 negatively regulates CXCL1, and CXCL1 then affects neutrophil recruitment, all under the control of *Dpc1*.

Relatively little is known about *Zfp30* except that it encodes an as yet uncharacterized C2H2 zinc finger protein that contains a Krüppel-associated box domain, signifying a role in gene repression via heterochromatin formation (Friedman *et al.* 1996). However, two features of the *Zfp30* eQTL provided important insights about *Zfp30* regulation. First, allele-specific gene expression data provided convincing evidence that the eQTL acts in *cis*. Second, haplotypes spanning the 5′ region of the gene, but not the 3′ region, were strongly correlated with *Zfp30* expression. More specifically, we found that expression levels in CC mice correlated strongly with the strain distribution pattern of 5′ region haplotypes: mice with haplotypes from NOD/ShiLtJ, NZO/H1LtJ, 129S1/SvImJ, or A/J founder strains had high expression; mice with haplotypes from C57BL/6J or WSB/EiJ founder strains had moderate expression; and mice with haplotypes from CAST/EiJ or PWK/PhJ founder strains had low gene expression.

These findings prompted us to seek to identify the specific variant(s) that regulate *Zfp30* expression. In this study, we utilized sequence data, epigenomic data, reporter gene assays, and electrophoretic mobility shift assays (EMSA) to interrogate the effect that specific variants have on *Zfp30* expression. In aggregate, our data point to a causal variant responsible for the modulation of *Zfp30* expression, and show that this variant alters transcription factor binding.

## Materials and Methods

### Cell culture

The MLE12 mouse lung epithelia cell line (generated from FVB/N mouse strain) was cultured in a 1:1 mix of Dulbecco’s Modified Eagle Medium and Ham’s F12 supplemented with 5% fetal bovine serum (FBS). The LA4 mouse lung airway cell line (generated from the A/He mouse strain) was cultured in Ham’s F12K medium supplemented with 15% FBS. Both cell lines were grown at 37° with 5% CO_2_. Twenty-four-well plates were seeded at a density of 120,000 cells per well for MLE12 or 125,000 cells per well for LA4 24 hr prior to transfection for luciferase assays.

### Dual luciferase reporter assay

A 500 bp genomic region of the *Zfp30* promoter surrounding a euchromatic region containing a variant of interest was amplified from five of the eight CC founder strains. Amplified candidate promoters were subcloned into the multiple cloning site upstream of the firefly luciferase gene in the promoterless pGL4.10 vector (Promega, Madison, WI) using a Gateway subcloning approach, and then verified by sequencing. MLE12 or LA4 cell lines were cotransfected in 24-well cell culture plates with 500 ng of candidate promoter constructs and 28 ng of *Renilla* control vector pRL-SV40 (Promega), using Lipofectamine 3000 (Life Technologies, Carlsbad, CA), according to manufacturer’s protocol. Transfected cells were cultured 48 hr before cell lysates were collected. Luciferase activity was measured using the Dual Luciferase Assay System (Promega). Firefly luciferase activity was normalized to *Renilla* luciferase activity as a control for transfection efficiency. Data are reported as the ratio of firefly to *Renilla* luciferase activity, and these data are provided in Table S1. Assays include four replicates for each candidate promoter and two technical replicates for each lysate collected. Luciferase assays were performed in each cell line at least two times, and results were consistent across experiments. Welch’s two-sided *t*-tests were performed to compare luciferase activity between promoter constructs.

### qPCR of luc2 and Renilla luciferase

mRNA expression of firefly and *Renilla* luciferase was assayed in MLE12 RNA samples collected 48 hr post-transfection with candidate promoters and pRL-SV40. iTaq Universal SYBR Green Supermix (Bio-Rad, Hercules, CA) was used in conjunction with *luc2* primers (Fwd: 5′-GTGGTGTGCAGCGAGAATAG-3′; Rev: 5′-CGCTCGTTGTAGATGTCGTTAG-3′) or *Renilla* luciferase primers (Fwd: TCACTATAGGCTAGCCACCAT-3′; Rev: 5′-CACTGCGGACCAGTTATCATC-3′). We validated the qPCR primers for efficiency by performing assays with serial dilutions of template and verifying the expected changes in Cq values. In subsequent assays, *Renilla* luciferase levels were used to normalize *luc2* Cq values, and fold change in expression of A/J and 129S1/SvImJ over C57BL/6J was calculated.

### Identification of genetic variants among CC founder strains

We utilized the Sanger Mouse Genomes Project variant database (Keane *et al.* 2011) (http://www.sanger.ac.uk/sanger/Mouse_SnpViewer/rel-1505) to identify variants within our cloned promoter region, which spans 29783622–29784093 bp on chromosome 7. These variants are shown in Table 1.

**Table 1:** *Zfp30* expression levels among CC founder strains and strain distribution patterns (SDPs) for 5′ region SNPs

Strain*^a^*	*Zfp30* Expression*^b^*	Relative Expression	Expression Group	rs32436680	rs258741331	rs31291744	rs211739374	rs230547036	rs252419650	rs578896492	rs581321876	rs239071968	rs51434084
A/J	7.72	1.86	High	C	T	C	C	G	C	T	C	G	G
NOD/ShiLtJ	7.65	1.77	High	C	T	C	C	G	C	T	C	G	G
NZO/H1LtJ	7.56	1.67	High	C	T	C	C	G	C	T	C	G	G
129S1/SvImJ	7.57	1.68	High	T	T	T	C	G	C	T	C	G	G
C57BL/6J	6.82	1.00 (Ref.)	Moderate	T	T	T	C	G	C	T	C	G	C
WSB/EiJ	6.69	0.91	Moderate	T	T	T	C	G	C	T	C	G	C
CAST/EiJ	6.48	0.79	Low	C	C	C	A	C	T	C	T	A	C
PWK/PhJ	6.42	0.76	Low	C	T	C	A	C	T	C	T	A	C

a The Sanger Mouse Genomes Project variant database was utilized for SNP data.

b Expression data (log2 units) based on data from Rutledge *et al.* (2014).

#### Site-directed mutagenesis:

Site-directed mutagenesis to change the rs51434084 allele in pGL4.10 constructs was carried out using the QuikChange II XL Site-Directed Mutagenesis Kit (Agilent, Santa Clara, CA) according to manufacturer’s protocol. Primers (Fwd: 5′-CCTCCAACCCCCTTCCCGTGACCAAGAGCTAGGGGCC-3′; Rev: 5′-GGCCCCTAGCTCTTGGTCACGGGAAGGGGGTTGGAGG -3′) were designed to mutate the position corresponding to rs51434084 from C to G.

#### EMSA:

Complementary 17 bp oligonucleotide probes centered on rs51434084 were purchased from (Integrated DNA Technologies, Coralville, IA) for use in EMSA experiments. Sequences for the moderate (C57BL/6J) and high (A/J) expression haplotypes, respectively, were as follows: Fwd: 5′-CCCTTCCCCTGACCAAGAG-3′; Rev: 5′-CTCTTGGTCAGGGGAAGGG-3′; and Fwd: 5′-CCCTTCCCGTGACCAAGAG-3′; Rev: 5′-CTCTTGGTCACGGGAAGGG-3′. Sets of complementary probes were ordered with and without biotin end-labeling (Integrated DNA Technologies). Complementary oligos were annealed into double-stranded DNA probes at a concentration of 2 pmol/μl by heating complimentary oligos to 95° in an annealing buffer (10 mM Tris, 50 mM NaCl; 1 mM EDTA pH 8.0) and cooling them to 25° in a thermocycler at a rate of 1° per minute. NE-PER Nuclear and Cytoplasmic Extraction Reagents (Thermo Scientific, Waltham, MA) were used to collect nuclear protein lysates from MLE12 cells. Protein concentrations were quantified using the Pierce BCA Protein Assay (Thermo Scientific).

EMSA experiments were performed using the LightShift Chemiluminescent EMSA Kit (Thermo Scientific) according to the manufacturer’s protocol. DNA–protein binding reactions contained 1× binding buffer, 1 μg poly(dIdC), 4 μg nuclear protein lysate, 200 fmol biotinylated DNA probe, and water, for a total reaction volume of 20 μl. Reactions were incubated at room temperature for 30 min prior to gel loading. Reactions including nonbiotinylated probes for DNA competition were incubated with 45-fold excess unlabeled probe for 25 min prior to addition of biotinylated probe. ZFP148 supershift reactions contained either 10 μg (Figure 4) or 20 μg (Figure S3) antibody incubated for 30 min prior to addition of biotinylated probe. Antibodies for ZFP148 (Fisher Scientific, Hampton, NH) and KLF4 (Millipore, Burlington, MA) were ordered for use in EMSA supershifts. DNA–protein complexes were separated on 6% DNA retardation gels (Life Technologies) using 0.5× TBE buffer (Life Technologies). Complexes were transferred to Biodyne B Pre-Cut Modified Nylon Membranes (Thermo Scientific) and UV cross-linked before chemiluminescent detection.

#### Transcription factor binding site prediction:

To identify transcription factor binding sites potentially affected by rs51434084, we used TRANSFAC (http://gene-regulation.com/pub/programs.html#match) and HOCOMOCO (http://opera.autosome.ru/perfectosape/scan/new) prediction tools. Based on EMSA results indicating enhanced binding of nuclear proteins to the C57BL/6J allele (*vs.* 129S1/SvImJ), we limited our search to putative transcription factor binding sites in which the C57BL/6J allele is predicted to bind the transcription factor better than the 129S1/SvImJ allele. We then performed literature searches on the initial list of candidates to prioritize any transcription factors with a known connection to regulation of neutrophil recruitment.

### Data availability

Luciferase reporter data are provided in Table S1. Original EMSA images for Figures 3 and 4 are provided as Figure S4 and Figure S5.

## Results

### Candidate regulatory region for Zfp30

We sought to identify the causal variant (or variants) responsible for the *Zfp30* *cis*-eQTL on chromosome 7 we previously identified in whole lung tissue from incipient CC lines, which we showed was associated with variation in CXCL1 and neutrophil counts in response to allergen challenge (Rutledge *et al.* 2014). In that study, we found that incipient CC lines with chromosome 7 QTL region haplotypes from NOD/ShiLtJ, NZO/H1LtJ, 129S1/SvImJ, or A/J strains had high *Zfp30* expression, mice with haplotypes from C57BL/6J or WSB/EiJ founder strains had moderate expression, and mice with haplotypes from CAST/EiJ or PWK/PhJ founder strains had low gene expression. This same pattern of expression was observed among CC founder lines (Table 1), indicating that the *cis*-eQTL is the major determinant of *Zfp30* expression.

To identify regulatory regions that may contribute to variation in *Zfp30* expression, we examined ENCODE data for euchromatic regions marked by DNAse I hypersensitivity near the 5′ region of *Zfp30* in mouse lung tissue, because our previous data indicated a strong correlation between 5′ region haplotypes and gene expression (Rutledge *et al.* 2014). We examined a region spanning 10 kb upstream through intron 1 and identified a ∼ 500 bp region of open chromatin near the putative *Zfp30* promoter and transcription start site (Figure S2), which contains 10 SNPs. An examination of the strain distribution patterns within the identified region of open chromatin at the *Zfp30* promoter revealed a single shared SNP (rs51434084) among the high expression strains and six shared SNPs among the low-expressing strains (Table 1). These variants were thus considered priority candidates that might affect regulation of *Zfp30* in a manner consistent with the three expression groups.

### Candidate regulatory region significantly modulates reporter gene activity in mouse airway epithelia cell lines

To interrogate the effect of this candidate regulatory region on *Zfp30* expression, we cloned the 500 bp putative promoter region from representative strains of each *Zfp30* expression group into a promoterless luciferase vector for use in luciferase reporter assays. More than one strain was chosen from the high and low expression groups to account for potential differences in gene expression due to variants in the region. Specifically, promoter regions were cloned from A/J and 129S1/SvImJ to represent the high expression group, from C57BL/6J to represent the intermediate expression group, and from CAST/EiJ and PWK/PhJ to represent the low expression group.

**Figure 1:**
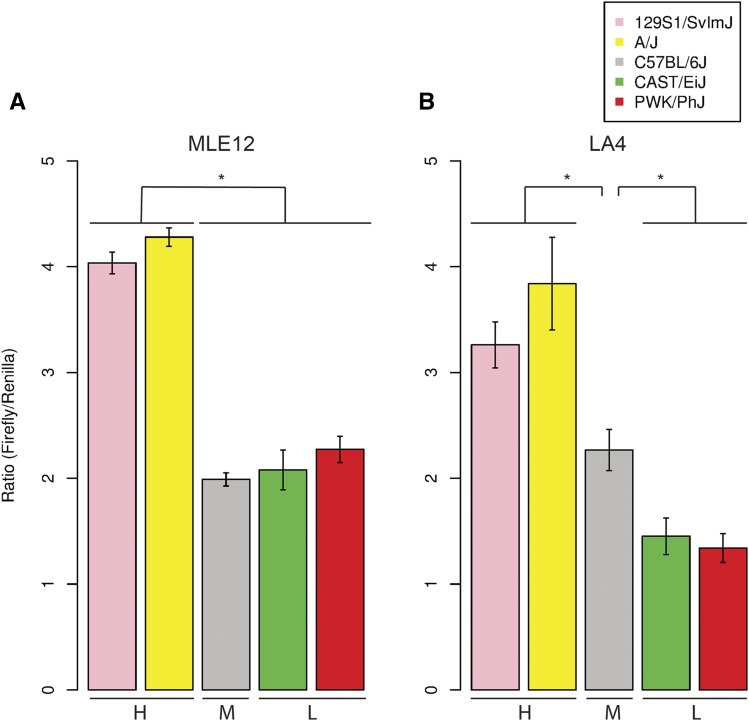
*Zfp30* promoter region haplotypes recapitulate *in vivo* patterns of gene expression in reporter assays. Dual luciferase reporter assays were performed, and firefly/*Renilla* ratios are shown. Reporter clones are grouped by *in vivo Zfp30* expression level: high (H), moderate (M), and low (L). (A) Results from assays in MLE12 cells recapitulate *in vivo* differences between moderate-expressing (C57BL/6J) and high-expressing (A/J and 129S1/SvImJ) strains, but not low-expressing (CAST/EiJ and PWK/PhJ) strains. (B) Results from assays in LA4 cells recapitulate *in vivo* differences between all three expression groups. * *P* < 0.05.

We then tested whether these promoter constructs recapitulate relative patterns of expression seen *in vivo*, using luciferase reporter assays in two mouse lung epithelia cell lines, MLE12 and LA4. Mouse lung epithelia cell lines were chosen because *Zfp30* expression was previously reported in this tissue. Additionally, we previously reported that knockdown of *Zfp30* using siRNAs in MLE12 cells led to increased CXCL1 secretion in response to LPS exposure, which is consistent with predictions based on *in vivo* data (Rutledge *et al.* 2014). Concordant with previously measured *in vivo* expression data (see Table 1), initial luciferase experiments in the MLE12 mouse lung epithelia cell line (Figure 1A) revealed a significant twofold difference in expression driven by promoters from high-expressing strains (A/J and 129S1/SvImJ) compared with lower-expressing strains (C57BL/6J, CAST/EiJ, and PWK/PhJ). No difference in luciferase activity was observed between the moderate-expressing (C57BL/6J) and low-expressing (CAST/EiJ and PWK/PhJ) promoter constructs in this cell line. This assay was also performed in the LA4 mouse airway cell line to compare results across differing genetic backgrounds. In LA4 cells, the expression difference between high and moderate expression groups was also evident, and at a similar magnitude. Additionally, CAST/EiJ and PWK/PhJ promoters produced significantly lower expression than C57BL/6J promoter (Figure 1B), at a magnitude roughly equivalent to the *in vivo* differences shown in Table 1. To verify that the differences we observed exist at both the protein and RNA levels, we measured *luc2* and *Renilla* luciferase mRNA levels in transfected MLE12 cells using a custom qPCR assay. The results, shown in Table S2, confirmed that the difference we observed in protein activity was also present at the RNA level.

We then focused our attention on the identification of SNPs that could explain the observed twofold expression difference between moderate and high *Zfp30* expressing promoters, for two reasons. First, the difference between moderate and high expression strains is much larger than the expression difference between moderate and low expression strains (Table 1). Second, in addition to our previous finding regarding the link between *Zfp30* expression and immune response in the lung (Rutledge *et al.* 2014), a previous report from a different research group indicates that the aforementioned expression difference is likely also the cause of contrasting response to *Streptococcus pneumoniae* infection (Denny *et al.* 2003).

**Figure 2:**
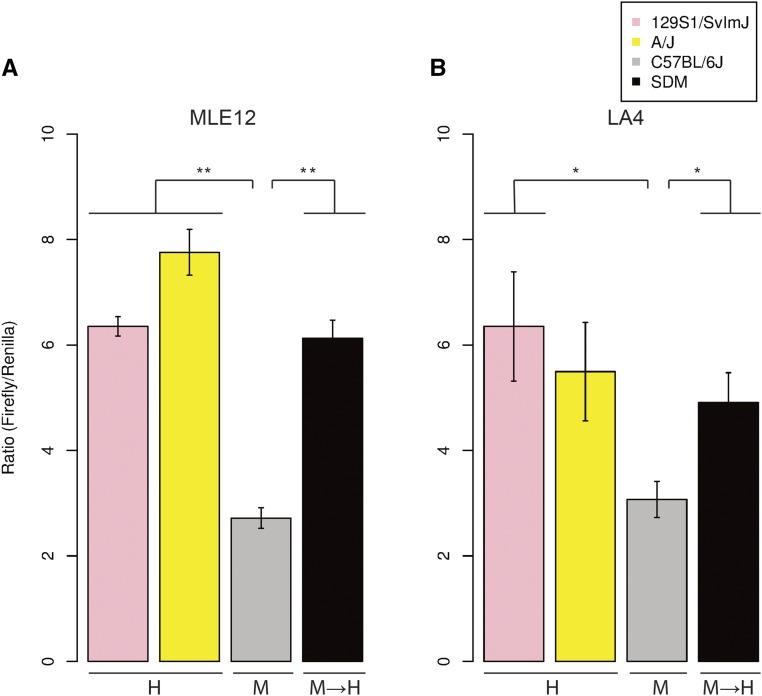
rs51434084 significantly modulates reporter gene expression. Dual luciferase reporter assays with haplotypes from A/J, 129S1/SvImJ, and C57BL/6J strains, as well as C57BL/6J haplotype with the rs51434084 C > G site-directed mutant (SDM) allele, were performed, and firefly/*Renilla* ratios are shown. Reporter clones are grouped by *in vivo Zfp30* expression as in Figure 1, with the addition of a haplotype generated by SDM of rs51434084 (M → H). (A) Results from assays in MLE12 cells show a significant increase in SDM construct compared with the C57BL/6J construct. (B) Results from assays in LA4 cells show a significant increase in SDM construct compared with the C57BL/6J construct. ** *P* < 0.001, * *P* < 0.05.

Within the cloned regulatory region, A/J and 129S1/SvImJ share only one variant, rs51434084. To test the regulatory effect of this SNP, we used a site-directed mutagenesis approach to modify a C57BL/6J promoter at this locus to match the allele of the high-expressing strains. The resulting promoter contains a single bp modification that results in a doubling of expression in both MLE12 (*P* < 0.001) and LA4 (*P* < 0.05) cell lines (Figure 2), matching the expression levels of A/J and 129S1/SvImJ.

### Variation at rs51434084 results in differential binding of ZFP148

**Figure 3:**
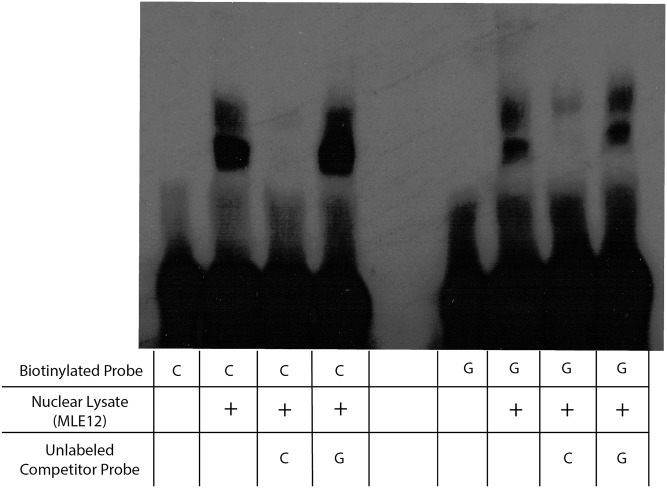
Electrophoretic mobility shift assays reveal differential binding of a transcription factor at the rs51434084 locus. Biotin-labeled 17-bp double-stranded DNA oligonucleotides containing contrasting rs51434084 alleles were incubated with 4 μg of MLE12 nuclear lysates. Unlabeled probes were incubated at 45× concentration of the labeled probes. The unmodified image is shown in Figure S4.

We then performed EMSA experiments to detect differential binding of transcription factors from MLE12 nuclear lysates to 17 bp probes mimicking either the C57BL/6J or 129S1/SvImJ allele at rs51434084 (Figure 3). Multiple DNA–protein complexes were observed for each probe, and each complex showed higher affinity for the C57BL/6J probe. Competition reactions with unlabeled probes revealed that the unlabeled C57BL/6J probe competed these interactions away with higher affinity than the unlabeled 129S1/SvImJ probe. Given that *in vivo* data and *in vitro* reporter assays both indicated that the C57BL/6J haplotype is associated with lower gene expression than the 129S1/SvImJ haplotype, we reasoned that the observed nuclear binding to the probe sequence containing rs51434084 is repressive.

To identify candidate proteins that participate in these DNA–protein complexes, we used the transcription factor binding site prediction tools TRANSFAC and HOCOMOCO to search for known mouse DNA-binding proteins predicted to differentially bind to the C57BL/6J and 129S1/SvImJ probe sequences. Multiple candidates emerged from this analysis (Table S3), and literature searches revealed that two candidates have established roles in immune regulation and have repressive transcriptional activity, rendering them priority candidates. ZFP148 (also known as ZBP-89, BERF-1, and BFCOL1) has been shown to regulate CXCL5, a chemokine similar to CXCL1 in function (Keates *et al.* 2001). KLF4 (also known as GKLF) was associated with endothelial barrier integrity and severity of lipopolysaccharide-induced lung injury (Cowan *et al.* 2010).

**Figure 4:**
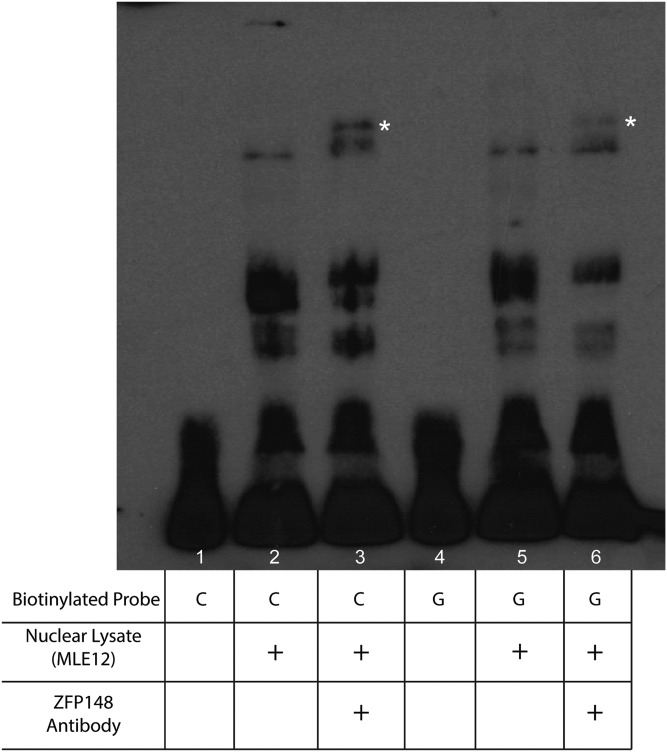
Electrophoretic mobility shift assay supershift reveals differential ZFP148 binding. Biotin-labeled 17-bp double-stranded DNA oligonucleotides containing contrasting rs51434084 alleles were incubated with 4 μg of MLE12 nuclear lysates. The supershift condition was incubated with 10 μg ZFP148 antibody. White asterisks denote the supershift band. The unmodified image is shown in Figure S5.

EMSA experiments with a supershift condition did not reveal a supershift with KLF4 antibody (data not shown). In contrast, addition of ZFP148 antibody produced a supershift (Figure S3), indicating that ZFP148 is among the DNA–protein complexes identified in Figure 3. We detected differential intensity of the supershifted band when probing with oligos representing the rs51434084 C or G alleles (Figure 4, lanes 3 and 6), accompanied by differential intensity of the lower band that is reduced by ZFP148 antibody addition (Figure 4, lanes 2 and 5). These results indicate that ZFP148 is among the nuclear proteins with higher affinity to the C57BL/6J probe.

## Discussion

Neutrophil levels in human airways correlate with asthma disease severity (Wenzel *et al.* 2003; Moore *et al.* 2014), poor response to inhaled corticosteroids (Green *et al.* 2002), and sudden-onset fatal asthma attacks (Sur *et al.* 1993). In COPD, neutrophil-to-lymphocyte ratio (NLR) is associated with disease state, and increased NLR is associated with longer hospital stays and higher readmission rates (Günay *et al.* 2014; Duman *et al.* 2015). Additionally, neutrophil extracellular traps are associated with increased airflow limitation among patients with COPD, and neutrophilic inflammation leads to decreased lung function (Quint and Wedzicha 2007; Hoenderdos and Condliffe 2013; Grabcanovic-Musija *et al.* 2015). In cystic fibrosis, neutrophil secretory proteins drive disease states and increase mucus production (Mackerness *et al.* 2008; Gifford and Chalmers 2014; Laval *et al.* 2016). In acute lung injury, neutrophils are a key component to lung damage, and their recruitment is controlled by complex chemokine networks (Grommes and Soehnlein 2011; Williams and Chambers 2014). Genes related to neutrophils and their recruitment are also upregulated in early sepsis-induced ARDS (Kangelaris *et al.* 2015). These findings indicate that increased understanding of neutrophil recruitment to the lung would have broad importance.

To gain insight into the regulation of neutrophil recruitment, we exploited a mouse model of asthma featuring neutrophilic inflammation. In the context of this model system, we identified *Zfp30* as a candidate regulator of CXCL1 levels and neutrophil recruitment (Rutledge *et al.* 2014). Here, we follow up this finding by identifying a causal variant for the variation in *Zfp30* expression, namely rs5143408.

Our reporter assays with *Zfp30* promoter constructs provided two main findings. First, this 500 bp region successfully recapitulated the twofold range of variation in *Zfp30* expression we previously characterized *in vivo* (Rutledge *et al.* 2014). Second, allele-swapping of rs5143408 using site-directed mutagenesis shows that this variant is the primary driver of the twofold expression difference between these strain groups. We also note that in our *in vitro* reporter assays, the expression pattern for CAST/EiJ and PWK/PhJ differed between the two cell lines we used (MLE12 and LA4). The expression pattern seen in the LA4 cell line (generated from the A/He mouse strain) matched mRNA expression results seen *in vivo*, but the pattern seen in MLE12 (generated from FVB/N mouse strain) did not. This suggests that these cell lines differentially express a repressor protein that downregulates expression of *Zfp30* in CAST/EiJ and PWK/PhJ. Future experiments will be directed toward identifying the causal variant(s) and protein(s) responsible for this modulation of *Zfp30* expression.

Our EMSA results indicate that ZFP148 is among the transcription factors that bind to the rs51434084 locus in the *Zfp30* promoter region and thereby regulate *Zfp30* expression. Thus, we propose that our initial model, in which *Zfp30* expression regulates CXCL1 and neutrophil recruitment (Rutledge *et al.* 2014), be expanded to include ZFP148. ZFP148 has a known role in the negative regulation *of Cxcl5*, a CXCR2 ligand similar to CXCL1, in human colonic epithelial cells (Keates *et al.* 2001). In combination with previous findings, our results suggest that ZFP148 downregulates both *Cxcl5* and *Zfp30*. These functions may seem contradictory at first, as *Zfp30* is proposed to downregulate *Cxcl1*, but decreased expression of *Cxcl5* has actually been linked to increased neutrophil recruitment into the lungs in an *Escherichia coli* pneumonia mouse model (Mei *et al.* 2010) through a mechanism involving Duffy antigen receptor for chemokines (DARC)–mediated sequestration of CXC chemokines. This mechanism results in a steeper gradient of CXCL1 and CXCL2 chemokines between vasculature and lungs. Thus, the function of ZFP148 proposed here could be cooperative with its known effect on CXCL5 levels.

ZFP30 is a C2H2 zinc finger protein with domains that suggest DNA-binding and KAP-1–binding functions. Wherever ZFP30 binds in the genome, a complex of proteins recruited by KAP-1 are predicted to induce heterochromatin domains and silence nearby genes (Friedman *et al.* 1996; Ryan *et al.* 1999; Schultz *et al.* 2002; Medugno *et al.* 2005). Specific binding sites for ZFP30 have not yet been identified, and additional work is needed to characterize the mechanism of ZFP30-mediated control of neutrophil recruitment. *Cxcl1* is a priority locus of interest; however, KAP-1/zinc finger protein complexes could have multiple binding sites and can act over tens of kilobases (Groner *et al.* 2010), so multiple genes may be affected.

This work contributes to an increased understanding of how *Zfp30* is regulated as a function of an allelic variant, which we previously showed was linked to neutrophils in the airways in a mouse model. Future work examining the relevance of the genes/proteins studied here, namely ZFP148 and ZFP30, in neutrophil chemotaxis in other model systems and in human patients is a critical next step to evaluating the biomedical importance of our findings. Additionally, identification and characterization of additional functional variants that impact neutrophil recruitment will further develop our understanding of the complex regulatory networks that drive disease severity phenotypes in both model systems and human patients with asthma, acute lung injury, cystic fibrosis, or COPD.

## Supplementary Material

Supplemental material is available online at www.g3journal.org/lookup/suppl/doi:10.1534/g3.117.300507/-/DC1.
