# Supplementary figures and images for: Differential Regulation of Zfp30 Expression in Murine Airway Epithelia Through Altered Binding of ZFP148 to rs51434084
Source: G3 (Bethesda). 2017 Dec 13;8(2):687–93. doi: 10.1534/g3.117.300507 (PMC5919737; doi:10.1534/g3.117.300507)

Figure S1.

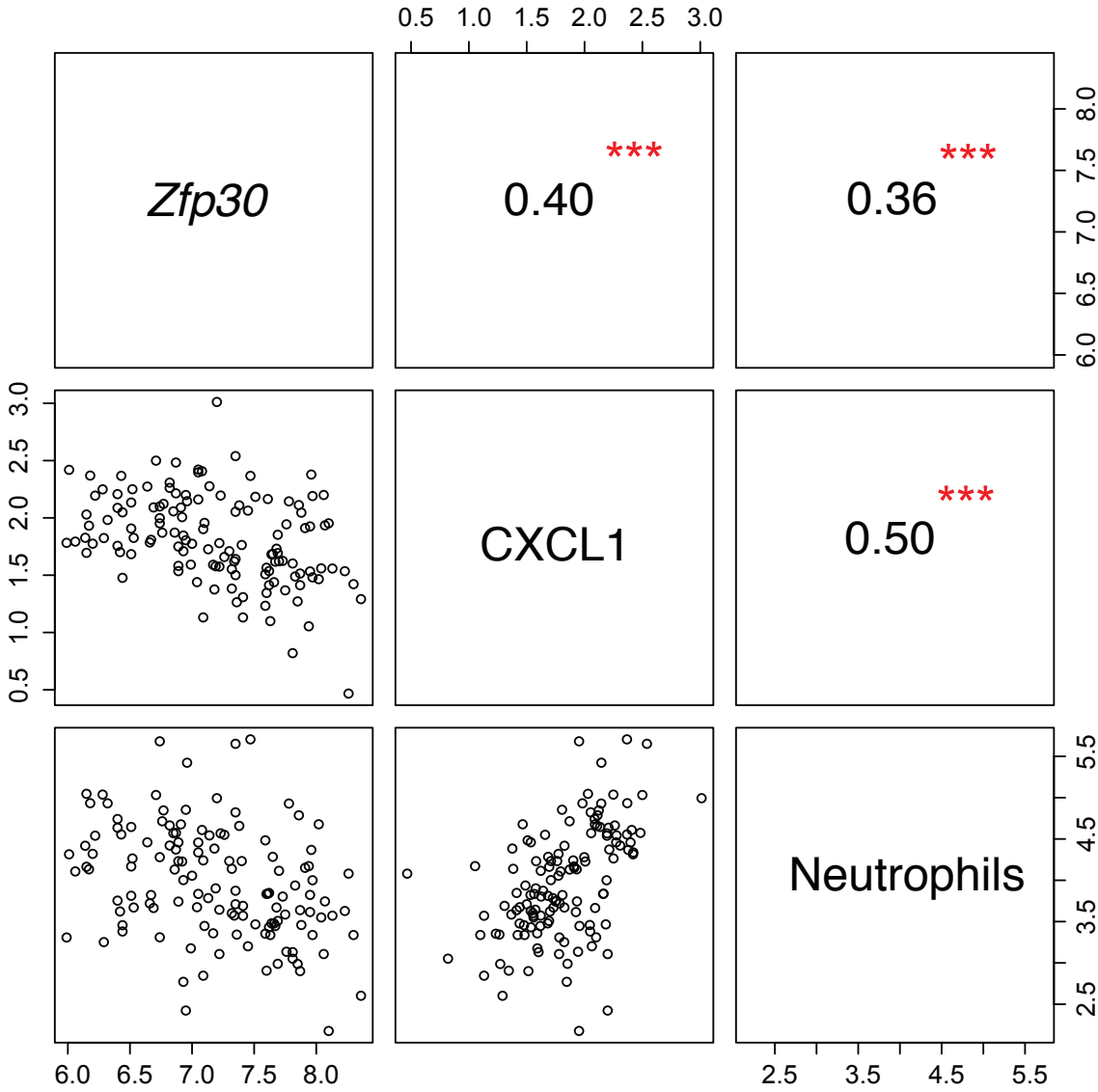

Supplement: Supplementary file 1 [file 687FigureS1.pdf]

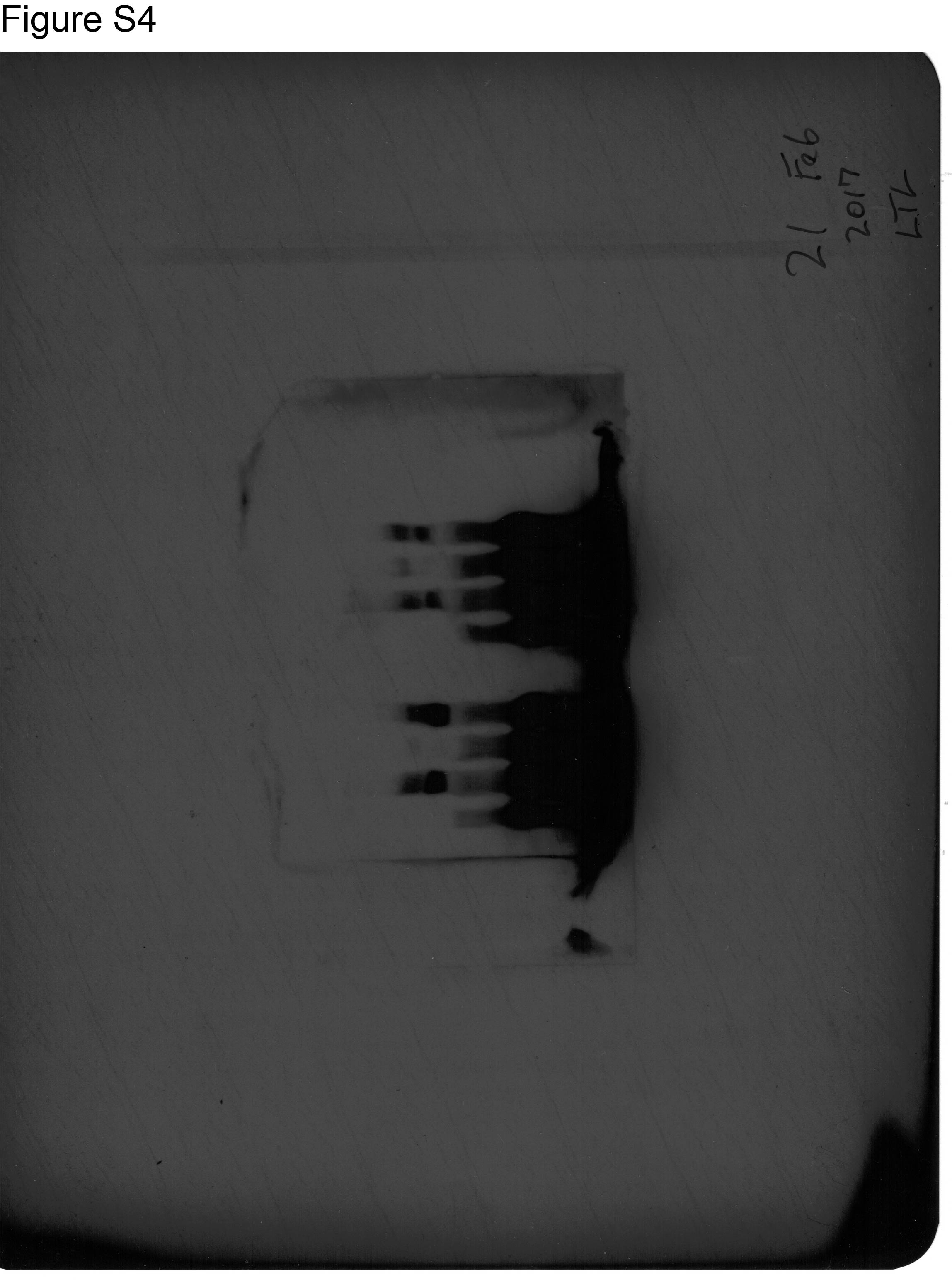

Supplement: Supplementary file 2 [file 687FigureS4.jpg]

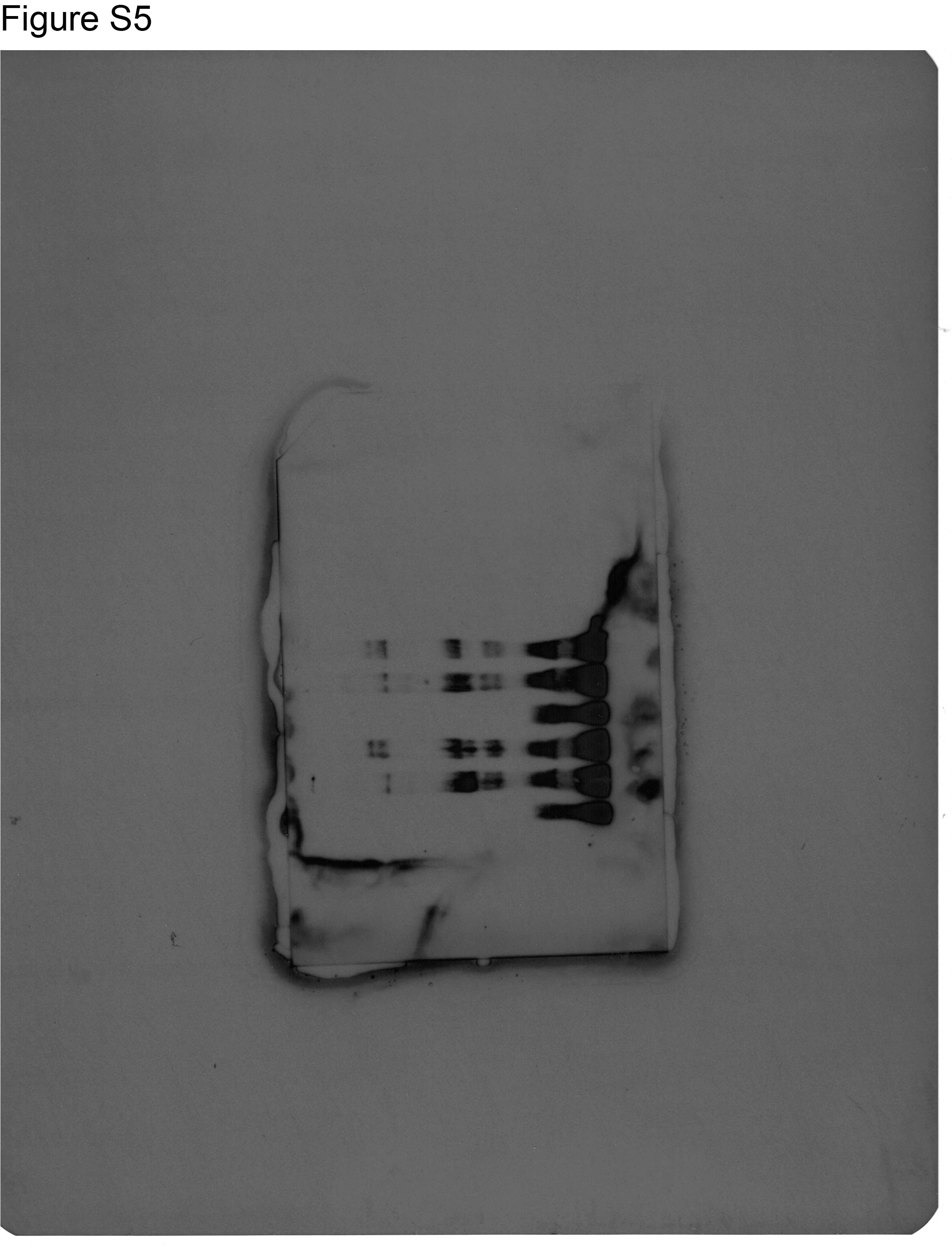

Supplement: Supplementary file 3 [file 687FigureS5.jpg]
