# Supplementary material for: Differential Regulation of Zfp30 Expression in Murine Airway Epithelia Through Altered Binding of ZFP148 to rs51434084
Source: G3 (Bethesda). 2017 Dec 13;8(2):687–93. doi: 10.1534/g3.117.300507 (PMC5919737; doi:10.1534/g3.117.300507)
Supplement: Supplementary file 5 [file 687TableS2.docx]

**Table S2. qRT-PCR reveals haplotype-dependent differences in reporter gene expression matching protein level measurements.**

| **Haplotype comparison vs. C57BL/6J** | **ΔΔCq** | **Fold Change** |
| --- | --- | --- |
| **A/J** | -0.834 | 1.783 |
| **129S1/SvImJ** | -0.828 | 1.775 |
