# Supplementary material for: Differential Regulation of Zfp30 Expression in Murine Airway Epithelia Through Altered Binding of ZFP148 to rs51434084
Source: G3 (Bethesda). 2017 Dec 13;8(2):687–93. doi: 10.1534/g3.117.300507 (PMC5919737; doi:10.1534/g3.117.300507)
Supplement: Supplementary file 6 [file 687TableS3.docx]

**Table S3. Transcription factor candidates for differential rs51434084 binding**

| Factor | Prediction Program* | Predicted motif (rs51434084 in bold) | Predicted enhanced binding to C57BL/6J allele? | Connection to immune regulation? |
| --- | --- | --- | --- | --- |
| MZF1 | TRANSFAC | TCCC**C**TGA | Yes |  |
| AP-2 | TRANSFAC | CTTCCC**C**TGACC | Yes |  |
| GKLF | TRANSFAC | CCC**C**TGACCAAGAG | Yes | Yes; PMID: 20724706 |
| NR0B1 | HOCOMOCO | CCTTCCC**C**TG | Yes |  |
| ZFP148 | HOCOMOCO | CCCTTCCC**C**TGACCA | Yes | Yes; PMID: 11559712 |
| INSM1 | HOCOMOCO | CTTCCC**C**TGACC | Yes |  |

*websites

TRANSFAC

<http://gene-regulation.com/pub/programs.html#match>

HOCOMOCO

<http://opera.autosome.ru/perfectosape/scan/new>
